# Career development for infection and immunity research in Uganda: a decade of experience from the Makerere University – Uganda Virus Research Institute research and training programme

**DOI:** 10.12688/aasopenres.13066.2

**Published:** 2020-08-17

**Authors:** Damalie Nakanjako, Flavia Zalwango, Pamela Wairagala, Fiona Luboga, Irene Andia Biraro, Victoria Diana Bukirwa, Mary Gorrethy Mboowa, Steve Cose, Janet Seeley, Alison Elliott

**Affiliations:** 1 Makerere University-Uganda Virus Research Institute Infection and Immunity (MUII), Uganda Virus Research Institute, Entebbe, Uganda; 2 Department of Medicine, School of Medicine, Makerere University, College of Health Sciences, Kampala, Uganda; 3 Medical Research Council/Uganda Virus Research Institute and London School of Hygiene and Tropical Medicine Uganda Research Unit,, Uganda Virus Research Institute, Entebbe, Uganda; 4 Global Health and Development Department, London School of Hygiene and Tropical Medicine, London, UK

**Keywords:** Academic careers, capacity building for research, Infection and Immunity, Immunology, bioinformatics, mentorship, collaboration, partnership, sub-Saharan Africa

## Abstract

**Background:** The Makerere University/Uganda Virus Research Institute (UVRI) Centre of Excellence for Infection & Immunity Research and Training (MUII) is a collaborative programme supporting excellence in Infection and Immunity (I&I) research in Uganda. Set up in 2008, MUII aims to produce internationally competitive Ugandan and East African I&I research leaders, and develop human and infrastructural resources to support research and training excellence. We undertook an internal evaluation of MUII’s achievements, challenges and lessons learned between 08-2008 and 12-2019, to inform programmes seeking to build Africa’s health research expertise.

**Methods: **Quantitative data were abstracted from programme annual reports. Qualitative data were obtained in 03-04/2019: a cross-sectional evaluation was undertaken among a purposefully selected representative sample of 27 trainees and two programme staff. Qualitative data was analysed according to pre-determined themes of achievements, challenges, lessons learned and recommendations for improvement.

**Results:** By 12-2019, MUII had supported 68 fellowships at master’s-level and above (50% female: 23 Masters, 27 PhD, 15 post-doctoral, three group-leaders) and over 1,000 internships. Fellows reported career advancement, mentorship by experts, and improved research skills and outputs. Fellows have published over 300 papers, secured grants worth over £20m, established over 40 international collaborations, and taken on research and academic leadership positions in the country. Key lessons were: i) Efficient administration provides a conducive environment for high quality research; ii) Institutions need supportive policies for procurement, including provisions for purchases of specific biological research reagents from international manufacturers; iii) Strong international and multi-disciplinary collaboration provides a critical mass of expertise to mentor researchers in development; and iv) Mentorship catalyses young scientists to progress from graduate trainees to productive academic researchers, relevant to society’s most pressing health challenges.

**Conclusions: **Sustainable academic productivity can be achieved through efficient operational support, global collaboration and mentorship to provide solutions to Africa’s health challenges.

## Introduction

From its inception in 2008 as a training programme, and transition in 2016 to a Centre of Excellence, the Makerere University/Uganda Virus Research Institute (UVRI) Centre of Excellence for Infection & Immunity Research and Training (MUII) has earned a reputation for building Ugandan capacity in infection and immunity (I&I) research. MUII, a partnership between Uganda’s leading health research institute (UVRI) and the country’s oldest and most prestigious university (Makerere University), collaborates with University of Cambridge, the London School of Hygiene & Tropical Medicine and other partners, and is currently funded through the African Academy of Sciences’ DELTAS Africa Initiative ^1, 2^. MUII aims to provide opportunities to bright young Ugandans to engage in Africa-led world-class research in I&I to provide solutions for Africa’s health challenges, and to contribute to improving global health. The current COVID-19 global crisis underscores the need for local expertise and infrastructure to support national responses to global health challenges within the local context.

MUII supports graduate trainees who include master’s and doctoral students and early career scientists [post-doctoral scientists within five years after PhD award and group leaders (post-doctoral scientists initiating research groups in a particular field of interest)] in I&I disciplines. In addition, MUII provides placements for undergraduate and new-graduate interns, including proactive support for individuals with disability, to develop interest and participate in biomedical research.

Unlike recent large-capacity building programmes that have focused on increasing the numbers and quality of medical graduates to support health care in resource-limited settings ^3, 4^, MUII supports development of local research scientists to harness cutting edge tools of immunology, molecular biology, genetics and bioinformatics to address the health challenges of tropical Africa ^1, 2^. Although PhD training has increased in the last decade through various collaborative programmes ^5^, there is limited experience and data on growth of academic careers from PhD graduates to independent scientists and leaders in science, policy and practice. This gap is emphasised by the scarcity of post-doctoral and research fellow positions in the establishment of several academic institutions in Uganda and the sub-Saharan region. We therefore found it necessary to systematically investigate and document the drivers of the success, the challenges and the key lessons from MUII that might be useful to other research capacity building programmes in Uganda and similar resource-limited settings, to further bridge the gap between doctoral degrees and academic research leadership, building public utility of local scientists to lead, inform and solve local problems within the global context.

## Methods

### Programme description

**Figure 1.**
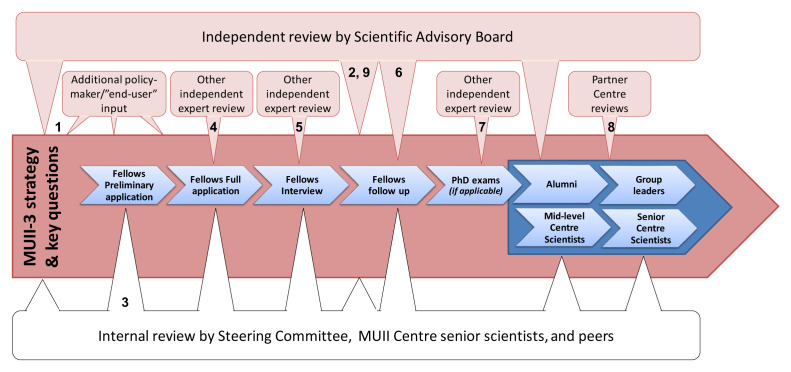
MUII Internal and External Scientific and Advisory Review Structures. *Advice and review is, and will be, provided by the Scientific Advisory Board (SAB) and by additional reviewers solicited among implementation colleagues (such as Ministries of Health, industry, non-governmental organisations) and expert colleagues from partner institutions (LSHTM, Cambridge, and collaborators. The large red arrow represents planning and progression of the programme as a whole; blue arrows represent recruitment and follow up of fellows and alumni. Numbers below explain those in the figure.* ***Advice on MUII ^3^ programme strategy*** *1. The SAB participation in planning for MUII3 through 2019 and 2020 AGM and SAB meetings. Additional input from policy-makers and other potential “end-users” of research has been initiated, and will continue during the planning and execution of MUII ^3^* *2. The SAB review reports and progress at each future MUII ^3^ AGM and guiding further direction* ***Advice on selection and progress of fellows*** *3. Preliminary applications long-listed by Executive and Steering Committee members* *4. Expert review for each full application, usually solicited from expert collaborators beyond the SAB* *5. Interview committees include SAB members and other reviewers, based on required expertise.* *6. Fellows’ progress reviewed at AGMs by SAB members* *7. High calibre external examiners and “opponents” for examinations* ***Advice for MUII’s emerging research leaders*** *8. Peer review and critical review by senior MUII scientists; independent expert review from scientists who are highly experienced in grantsmanship at LSHTM and Cambridge.* *9. SAB members and other international collaborators provided mentorship for MUII fellows.*

MUII supports a centre of excellence in I&I research and training, with a secretariat based at UVRI. The secretariat comprises the Director, Centre Manager, finance, administration, procurement, monitoring and evaluation, and communication teams which work in close collaboration with UVRI government staff. The secretariat implements, and monitors activities and processes that are agreed upon by the executive committee. The MUII secretariat also organised regular meetings which made it easy for people to be accountable not only for their time but also to their set goals. The centre is governed by an executive committee (EC), chaired by the Director, with representation from each category of trainees (master’s, PhD and post-doctoral) to guide and the implementation of the programme. The EC provides guidance to the secretariat in the execution of program activities. The programme has a Scientific Advisory Board (SAB) consisting of local and international experts in I&I who help to review all programme activities. A SAB member usually chairs fellowship selection committees; and SAB members attend the Annual General Meeting, participating in review of fellows’ progress, programme goals, activities, outputs and outcomes ( Figure 1).

MUII also employed scientific staff in areas requiring intensive support and development (initially a senior, expatriate immunologist; subsequently a more junior Ugandan immunologist (recently graduated from an American university); both at first full-time, later part-time. These supported initiation of core training in immunology, and subsequently contributed to development of Makerere’s master’s degree in immunology and clinical microbiology, and the development of a new department of Immunology and Molecular Biology, chaired by a MUII post-doctoral alumnus. Similarly, MUII has supported a lead scientist in bioinformatics, first as a post-doctoral fellow, subsequently as a part-time staff scientist. MUII offered training in research methods, data analysis, use of data analysis software packages, grant writing, report writing, presentation skills, among others.

MUII functions through core implementation teams called Impis (from the Zulu word for a band of warriors) which provide support for the fellows’ projects. The Impis include Immunology, Bioinformatics, Fellowships, Mentorship, Monitoring and Evaluation, Internships and Diversity, as well as Community Engagement ^1, 2^. In addition to fellowships, MUII provides opportunities for endeavour awards to allow innovative interventions and visiting scientists awards to allow fellows to invite or visit international experts in specific fields of interest to build research expertise. An executive committee chaired by the Director, with representation from each category of trainees (master’s, PhD and post-doctoral) and from each Impi, manages the implementation of the programme, and provides guidance to the secretariat in the execution of program activities.

### Evaluation design and participants

We reviewed programme monitoring and evaluation data, including annual reports (2008–2019), for quantitative data on number of trainees and outputs including publications and research grants. Additionally, in a cross-sectional study, qualitative evaluation was conducted in March and April 2019 by F.Z., a social scientist at the medical research council (with MSc in Gerontology) independent of the MUII programme. A representative sample of 27 trainees (out of 50 then available), and two centre staff was purposefully selected. The purposive representative sample considered representation of five categories of fellows: Masters, PhD, post-doctoral and group-leader fellows, and honorary fellows (not directly funded by MUII, but registered under MUII, participating in fellows’ meetings and training activities, and sometimes receiving small travel or equipment grants). The interviewer introduced herself to all interviewees on email, highlighted the purpose of the interview and made suitable appointments. All participants contacted accepted to participate in face-to-face interviews during working hours. The interview was attended by the interviewer, interviewee and two media staff to audio and video record. Skype interviews were conducted with participants unavailable for face-to-face interaction. Each interview lasted at least 45 minutes.

### Qualitative data collection

In-depth interviews (IDI) were conducted with the fellows and key informant interviews (KII) with staff, by using a pre-tested topic guide (see *Extended data*) to explore experiences from the MUII programme over the last 10 years. The guide was designed to obtain insights into achievements (both for the fellow and the institution), successes and career progression of trainees, growth in institutional I&I research capacity, sustainability, challenges, lesson learnt, recommendations for improvements and sustainability of the impact beyond the programme. We also included questions on new leadership roles and mentorship experience that fellows have been involved in after participating in the MUII programme.

### Qualitative data management and analysis

Qualitative interviews and Skype calls were audio-recorded and later transcribed verbatim. These data were underpinned on content analysis, coded by F.Z., and analysed thematically using a framework that was informed by the study objectives and preliminary findings, according to pre-determined themes of achievements, challenges, lessons learned and recommendations for improvement. The framework was for coding the study data with constant comparison across the team to ensure consistency. The coding of data was managed using NVivo 12.

### Ethical review and consent

The protocol for the qualitative evaluation was reviewed by the Research Ethics Committee of the Uganda Virus Research Institute, which gave a waiver of written consent and approval to publish these routine program data (REF GC/127/19/03/699). Participants gave verbal consent to participation in the interviews and the use of the material from the interviews, including quotes, in this paper. Permission was sought from participants whose quotations appear in the paper.

## Results

Results are presented under the major emerging themes which were; achievements, successes and career progression of trainees, growth in institutional I&I research capacity, sustainability, challenges, lesson learnt, and recommendations for improvements.

### Achievements

MUII’s principal achievements comprise a) the successes and career progression of trainees, b) growth in Infection and Immunity (I&I) research and training capacity at MUII partner institutions in Uganda, and c) establishment of sustainable international collaborations for young Ugandan scientists.

**Figure 2.**
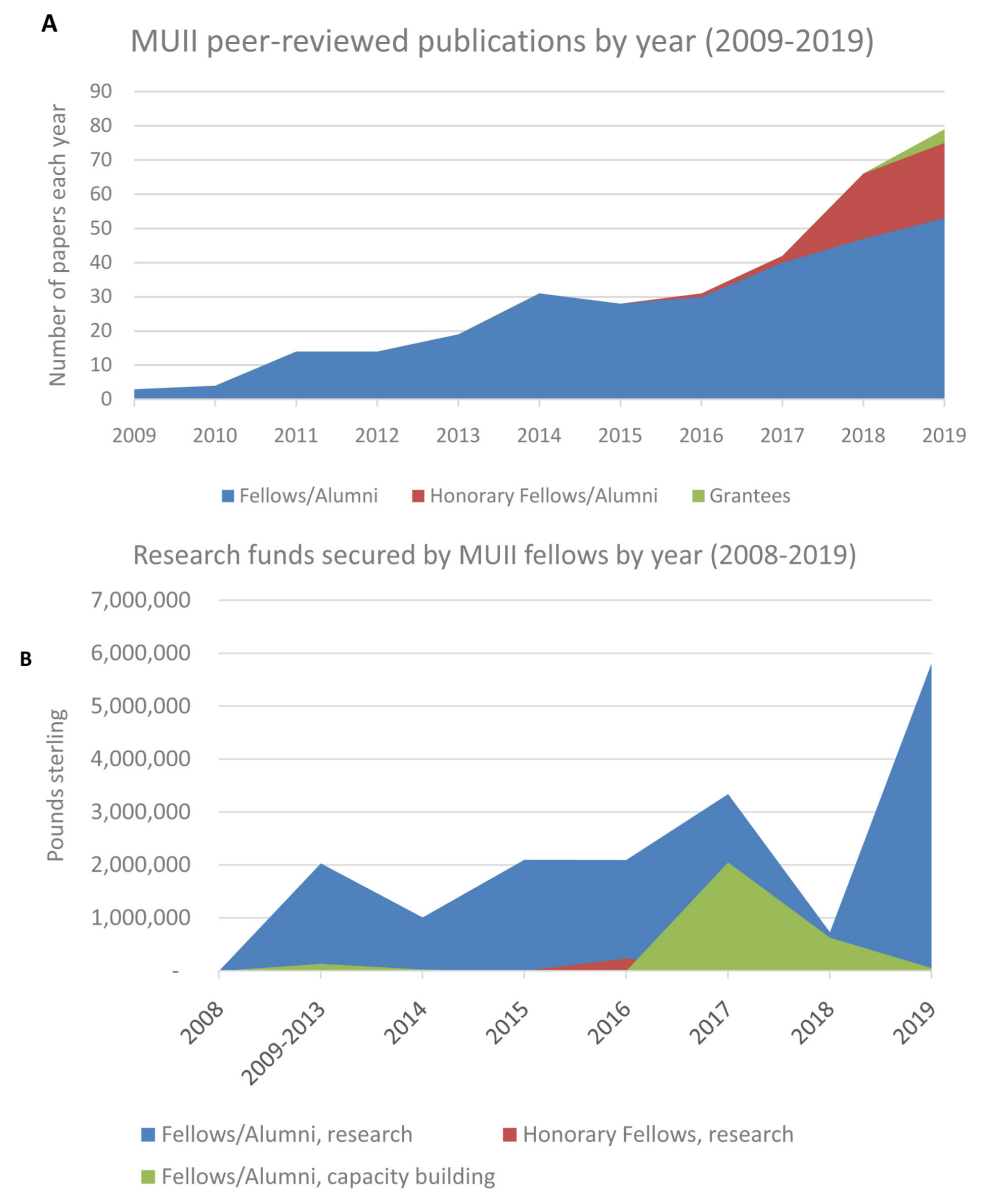
Research outputs by MUII fellows between January 2008 and December 2019: **A** shows peer-reviewed publications and **B**) shows amount of research funding received.

***Successes and career progression of trainees***. Between January 2008 and December 2019, MUII supported 68 fellowships including 23 master’s fellows, 27 PhD fellows, 15 post-doctoral fellows and three group leaders in I&I research. Of these, 53 were directly funded by MUII, while 15 were honorary fellows ( Table 1). By December 2019 these fellows, since joining MUII, had published over 300 peer-reviewed publications and received over 20 million Pound Sterling worth of additional funds for research ( Figure 2). These were all desired outputs of the programme demonstrating the achievement of the goal, “ *...to allow the Fellows* to *develop into independently funded research leaders*” (EMEL028, M, KII, Centre Staff). Other members of staff commented that seeing alumni ‘take off’ and become independent was a clear mark of success.

**Table 1.** Infection and Immunity research trainees between January 2008 and December 2019.

	MUII- funded	Honorary	Total	Female gender N (%)
Group Leaders	3	0	3	2 (67)
Post-docs	15	0	15	7 (47)
Doctoral students (PhD)	17	10	27	14 (52)
MSc students	18	5	23	11 (48)
**Overall**	**53**	**15**	**68**	**34 (50)**

All MUII alumni have taken on academic leadership roles including Principal Investigators, Heads of Departments, Deans of Schools, supervisors of graduate students, and mentors of other emerging scientists. MUII also supported over 1000 internships at UVRI and exposed over 5000 high school students to scientific research through “Open Days” at UVRI.

***Growth in I&I research at MUII partner institutions in Uganda***. Before MUII was launched, there were few facilities for immunology research at Makerere University. Starting with a small immunology laboratory under the Department of Microbiology, MUII contributed to human, technical and physical infrastructural development in I&I capacity. MUII supported renovations to improve space and equipment in the Immunology laboratory, as well as salary support for two immunologists to catalyse growth. Growth occurred, as evidenced by development of new Masters’ and doctoral training programmes in Immunology and Clinical Microbiology and in Genomics and Bioinformatics (with increasing numbers of applicants), with MUII alumni as heads of departments, supervisors and investigators in multi-disciplinary Infection and Immunity research ( Table 2).

**Table 2.** Growth in Infection and Immunity (I&I) Research at partner institutions Uganda Virus Research Institute and Makerere University.

MUII contribution	Makerere University	Uganda Virus Research Institute
**Research support**		
Infrastructural development	Laboratories within College of Health Sciences and College of Veterinary Medicine, Animal Resources and Biosecurity Computer laboratory, College of Natural Sciences	Training building with offices, meeting rooms and conferencing facilities and training laboratory IT equipment and software for trainees Financial systems equipment and software Support staff salaries and training
Laboratory capacity	Technician Support and equipment to translational lab at Infectious Diseases Institute Technology transfer by fellows training abroad to utilise acquired skills upon return to parent institution	Training Laboratory management Laboratory equipment Technology transfer by fellows training abroad to utilise acquired skills upon return to parent institution
Infection and immunity training programmes	Developed short course: Immunology in the tropics Contributed to start of MSc Immunology and Molecular biology Video-conferenced expert seminars Supported one immunologist Support to MSc and PhD in bioinformatics	Strengthened bioinformatics core with masters, doctoral and post-doctoral trainees Supported one immunologist Supported one molecular biologist/ bioinformatician
Student supervision	MUII trainees (MSc &PhD) were supervised by Makerere University faculty and collaborators MUII alumni continue to supervised MSc &PhD students	MUII trainees (MSc &PhD) were supervised by UVRI faculty and collaborators Supported one statistician to provide fellows’ statistical guidance
**Leadership**		
Research leadership	MUII alumni are now heads of departments, Deans, and leaders of professional association boards	MUII alumni are now key investigators and collaborators on institutional and collaborative projects
Developing collaborations & networks	All PhD, post-doctoral and group-leader fellows have travel support for collaboration Competitive Travel awards for programme staff and trainees Symposia and seminars	All PhD, post-doctoral and group-leader fellows have travel support for collaboration Competitive Travel awards for programme staff and trainees
Engagement with research participants & policy makers	All fellows receive funds for engagement	All fellows receive funds for engagement Open Days hosted at UVRI
**Programme** **organisation**		
	Support to existing schedules of supervisors’ and doctoral meetings in line with directorate of research and graduate training Support trainees to adhere to institutional research regulatory processes Internal monitoring of research	Administrative structure (secretariat) Executive Committee with representation of all the cadres, Masters, post-docs, PhDs Regular progress review meetings Monitoring & Evaluation Overarching Scientific Advisory Board of International experts in I&I

“ *Seeing these young Ugandans that have an interest in immunology, seeing that interest develop, bud and flower into a full-blown passion for immunology and seeing them start thinking about their research in immunological terms and to start to win grants in immunology! That for me is success*” (EMEL028, M, KII, Centre Staff)

***Sustainable training and research collaborations***. Through MUII, fellows created and consolidated collaborations and networks with over 40 institutions globally. Illustrative examples are listed in Table 3. These institutions have supported training in fields such as genetics, antimicrobial resistance, systems biology, bioinformatics, metagenomics, molecular and cell biology, vaccine development, and biomarker research ( Table 3): fields in which world class expertise was limited in Uganda. In addition, these collaborations have helped to position fellows to achieve recognition internationally:

**Table 3.** An illustrative sample of local and international collaborations with MUII between January 2008 and December 2019.

Collaborating institution (name and country)	Area of focus	Roles/contribution to MUII trainees
Uganda Virus Research Institute, Uganda	Laboratory skills	MUII programme host, statistics and bioinformatics core
Makerere University College of Heath Sciences, Uganda	Immunology, Bio-informatics, clinical care, patient cohorts	Masters and doctoral Programmes
Makerere University College of Natural Sciences, Uganda	Mathematical modelling	Trainees, mentors and dry lab space
Makerere University College of Veterinary Medicine, Animal Resources and Bio-Security, Uganda	Laboratory strengthening Immunology	Trainees, mentors and wet lab space
Rakai Health Sciences Project, Uganda	HIV pathogenesis and phylogenetics	Community cohorts and wet labs
Cambridge University and Cambridge Africa programme, UK	Metagenomics Genetics of pre- eclampsia	HLA typing, collaborative grant applications Training seminars
London School of Hygiene and Tropical Medicine, UK	Infection and Immunity	Mentors, statistics core and Scientific advisory board membership
Gladstone Institute of Vaccine Immunology, San Francisco, USA	HIV pathogenesis	Laboratory skills training and mentorship
The Vaccine and Gene Therapy Institute, Florida, USA	Systems biology	Laboratory skills training, workshop facilitation and mentorship
Case Western Reserve University, Ohio, USA	Systems biology	Laboratory skills training, workshop facilitation and mentorship
The Jenner Institute, Oxford, UK	Tuberculosis Vaccinology	Laboratory skills training and mentorship
Addenbrooke’s Hospital, Cambridge, UK	Genetics of pre-eclampsia	Laboratory skills training and mentorship
University of St. Andrews, UK	Antimicrobial resistance	Laboratory skills training and mentorship
University of Edinburgh, UK	Bioinformatics	Training and mentorship
PANGEA Network	Bioinformatics	Training and mentorship
Imperial College London, UK	Bioinformatics	Training and mentorship
Sanger Institute, UK	Metagenomics	Training and mentorship
Leiden University Medical Center, Netherlands	Parasite immunology and vaccines	Laboratory skills training and mentorship
University of Geneva Human Protein Lab, Switzerland	Plasma staging biomarkers	Laboratory skills training and mentorship

“ *…by linking me to Professors in the United Kingdom, it has really expanded my horizon because with them we have been able to compete at a bigger stage…Right now my light is shining because of this bigger stage that the collaboration has enabled me to get into. So it has really been very instrumental.*” (EMEL015, M, IDI, Group Leader).

### Challenges

However, interviewees reported several important challenges encountered.

***Procurement***. Delay in procurement of biological research supplies was reported as a critical challenge. Most government procurement systems did not have provision for direct orders from international suppliers of reagents specifically used in published immunology protocols, without the bureaucracy of local agents. This was a bottleneck to timely implementation of research and many fellows relied on international laboratory placements to complete their work. One fellow commented: “ *I spent most of my time in the lab overseas to avoid delays because that is where I could get my supplies continuously delivered almost immediately from the manufacturing company.*” (EMEL004, F, IDI, Group Leader). Others mentioned the need to place orders directly with manufacturers to avoid local agents and procurement delays.

Nevertheless, the procurement teams at UVRI and Makerere worked together to understand the issues affecting the researchers and did their best within the allowable clauses and limitations in policies for procurement of research reagents and equipment.

***Time management***. A common challenge for fellows was to meet deadlines, and many mentioned that they had to balance research with their clinical and teaching responsibilities: “ *One still has to perform their roles as a clinician, teacher, researcher, Head of Department, wife, mother, etc.*” (EMEL001, F, IDI, post-doc). Time pressure increased as fellows advanced in their research career.

***Limited pool of clinical immunology and bioinformatics experts***. Some fields had very limited local capacity for application of immunology and bioinformatics tools to address challenging threats: for example, the field of maternal health for the challenge of pre-eclampsia. MUII responded to this challenge through the support of international collaborators ( Table 3). Some of these collaborative efforts have resulted in lasting collaborations between fellows and supervisors, for example:

“ *The collaboration with Cambridge started with a PhD student and her mentor but now lots of other people involved in the health arena from Cambridge are working with her department. That is the ideal way we would like to see collaborations grow.*” (EMEL029, F, KII, Centre Staff).

***Uncertain sustainability of active research programmes beyond MUII***. Maintaining active research beyond their MUII funding was a challenge for fellows, with the prospect of attracting funding appearing quite daunting: “ *I need real significant funding which can maintain a group or team of six people for three years. I also need support in terms of someone commenting on the proposal or application and being connected to people of similar interests*.” (EMEL009, M, IDI, post-doc).

However, many fellows appreciated that this challenge was to be expected and competitive resource mobilisation is part of being a research scientist. To address this challenge, MUII organised skills-building meetings for fellows including grant writing and peer-review to increase the competitiveness of applications. In addition, MUII supports a mentorship programme where fellows can be linked to experts in their field.

### Lessons learned regarding key contributors to success

Interviewees shared key lessons learned in capacity building for research in resource-limited settings.

***Programme administration and organisation***. Good operational management was key to success of the training and research programme ( Figure 1 and Table 2). The distribution of roles to improve efficiency and the monitoring and evaluation functions helped the fellows to adhere to research regulations and programme timelines for master’s and doctoral students. Having an executive committee (EC) with representation from the different stakeholders including trainees and training institutions helped to think through most challenges and provide clear solutions within the context. Similarly, having a secretariat to follow through the implementation of EC recommendations helped the program to keep track of any internal or external influencers of trainees’ progress. Many of the fellows were grateful for the support they had received to enable them to graduate.

“ *MUII has had 100% success of [Masters] graduation on time. The programme pays attention to the Fellows. I think it has been excellent in terms of mentorship of the Fellows...*” (EMEL005, M, IDI, master’s).

***Multi-disciplinary collaboration within Uganda between leading academic and research institutions***. MUII showed that it was feasible to develop and sustain collaborations between UVRI and the Makerere Colleges of Health Sciences; Veterinary Medicine, Animal Resources and Biosecurity; Natural Sciences; and Computing and Information Sciences ( Table 3). This collaborative infrastructure was appreciated by fellows as a strength:

“ *…I applied [for the Group Leader Position] because I thought it would really give this cross multi disciplinarity, not just being at Makerere University, but also interacting with other research institutions in Uganda. UVRI is a premiere research institution in this country. So I thought it would be an opportunity for me to liaise with other scientists…*” (EMEL015, M, IDI, Group Leader).

***Procurement of research reagents and equipment***. Public academic and research institutions need to be supported to foster active framework agreements with international manufacturers of biological research reagents to minimise time delays created by looking for local agents who do not routinely import items that are specific to biological laboratory experiments and innovations.

***Mentorship***. Supporting young researchers to progress through MSc, PhD and post-doc programmes and subsequently to develop as group leaders to mentor other upcoming scientists and maintain the pipeline has been greatly valued.

“ *During the ten-year period, there were opportunities for MUII graduates at any level to apply (competing with other non-MUII applicants) for available MUII opportunities at the next level of career development. ‘I was privileged to have a very good supervisor and mentor who gave me time, gave my applications time and gave my research ideas time. So that I attribute to mentorship.*” (EMEL001, F, IDI, post-doc).

***Sustainability of I&I research***. Most fellows and graduates of the MUII programme have been able to win career development and research grants to sustain their research groups. Fellows, however, mentioned their need for more technical support to respond to funding opportunities as well as additional training in community engagement, leadership, and data management.

“ *I think we’ve been a pretty good example across the whole DELTAS [funding programme] network in getting out early PhD Fellows and they are in a position of getting their own research grants, they are heads of departments, deans of faculties and they are running their own profile research. I think that is where the sustainability comes in.*” (EMEL028, M, KII, Centre Staff).

Similarly, the MUII alumni that have grown professionally to take academic and research leadership positions are nurturing a culture that fosters high quality research in I&I research and are already mentoring other upcoming scientists.

“ *We have had a PhD funded fellow who is now working as Dean at the Vet School. We have a Group Leader who is the Dean of the Medical School. We have another Group Leader who has become a head of Department at Makerere University. So I think through them and other MUII alumni in other influential areas at Makerere University would make MUII and other research collaborations more sustainable.*” (EMEL029, F, KII, Centre Staff).

“ *…I have six PhD students that I supervise and mentor but in addition to that I mentor many other gynaecologists, even students outside my department…I mentor over 20 [students] because every time people come to consult me on different things.*” (EMEL018, F, IDI, Group Leader).

## Discussion

The MUII capacity-building programme in I&I has demonstrated ten years of high quality training in emerging tools of immunology and bioinformatics to answer locally generated questions to address gaps in knowledge and practice in the dominating infections of HIV, tuberculosis, malaria and its complications, helminths and emerging viruses, as well as the rising burden of non-communicable diseases including asthma, diabetes, hypertension and pre-eclampsia ^6– 32^. Through the spectrum of fellowships provided, from the most junior of undergraduate interns to master’s, doctoral, post-doctoral and group-leader awards, MUII has provided mentored research training opportunities to foster growth of academic research careers. With the engagement of scientific and non-scientific community, MUII scientists disseminated their science, its relevance in the local context and exposed it to over 5000 high school students (tomorrow’s scientists). The impact has been demonstrated by the programme monitoring and evaluation indicators that include, among others, peer-reviewed publications, additional funding for research and capacity building obtained by trainees, emergence of multi-disciplinary research groups, increasing applicants to the new MUII-supported masters’ and doctoral programmes in immunology and bioinformatics, and number of active local and international collaborations.

Unlike capacity-building initiatives that have focused on increasing the number of medical graduates for health care ^3, 4^ and doctoral training in various fields ^5, 33^, MUII has focused on developing experts in I&I - a major gap that slackens local biomedical responses to emerging infections in Africa. In addition, MUII has embraced the challenge of technology transfer of new immunology and bioinformatics techniques with the aim that MUII trainees will be empowered to lead novel initiatives like developing vaccine or therapeutic targets for emerging diseases. In a modest way, MUII tried to mimic what developing an academic research career would entail in high-income settings, where a developing research group requires fellowship schemes to kick-start scientists, PhD students and post-docs to be part of novel ideas and publications, technicians to support high-throughput equipment, dry laboratory scientists for genomic analyses and mathematical modelling ^2^. We recommend monitoring of equipment booking schedules to use wet laboratory equipment for immunology and molecular biology assays, abstracts presented at relevant local and international meetings, meetings held by our scientists with community, policymakers and other relevant stakeholders to demonstrate productivity and utility of the scientists. These are all critical for the next level of development of science in Africa. Improved management strategies are needed to tackle the epidemiological change facing Africa due to urbanisation and changes in lifestyle like the emerging burden of non-communicable diseases (NCDs) in Africa ^2^. Appropriate diagnostics, treatment and vaccines are needed in the face of unprecedented emerging pandemics, such as the current COVID-19 crisis. Locally generated evidence is critical to local management of such global pandemics; with examples of fast-tracked clinical trial data emerging from China and USA ^34, 35^ and the reliance of countries on their leading scientists ^36^ to inform national and global interventions. There is need for clinical trials within the local and cultural context of communities and health system. For example, it is not obvious that host responses to hydroxychloroquine and azithromycin as COVID-19 trial therapies in America would be similar to those in sub-Saharan Africa where both drugs have been used commonly for endemic malaria and atypical pneumonias, or that vaccines developed in high-income settings will be equally effective in Africa.

The MUII experience is unprecedented because of the previous absence of local advanced training programmes (MSc and doctoral level) in immunology and bioinformatics to advance locally trained scientists in I&I research. It is important to note that growing I&I scientists for public utility and creating a culture of mentored research opportunities for junior and mid-level scientists may take several years to decades; and even longer for institutions to attain critical mass of scientists with a culture of mentoring. Therefore, the lessons learned over the decade of implementation of the programme, presented here, are noteworthy to inform ongoing and future capacity building programmes on the African continent.

We have learned the need for a dedicated secretariat to support training programmes with communication, tracking, and evaluation to ensure timely completion of programme milestones. It is also critical for training programmes to provide technical expertise where it is needed for the success of trainees. MUII supported two immunologists to be part of the new department of Immunology and Molecular Biology that is chaired by a former MUII post-doc. Similar input was previously illustrated by an ongoing capacity building programme for cancer research that protected support staff time of approximately 0.25 to 0.5 full-time equivalents contributed to the success of the programme ^37^. During the regular programme capacity building meetings, fellows would critique each other’s work, which greatly improved the quality of proposals and presentations. Fellows also had opportunities to participate in different workshops and conferences. MUII fellows were prepared to become better mentors, and many of them acquired peer mentees, as previously noted that peer mentorship greatly supported in-country capacity building initiatives ^37^. At the regular progress meetings fellows discussed bottlenecks in their progress such as delayed procurement of specific reagents and equipment that required direct importation from manufacturers abroad. Both MUII institutions (UVRI and Makerere) still need support to develop and implement efficient policies that reduce the turn-around time for research reagents and equipment orders, many of which are expensive sole supplier items because of previously optimised protocols and equipment ^38^.

MUII programme increased trainees’ networking opportunities through the large pool of multi-disciplinary scientists and through several collaborations established as well as the visiting scientist awards where fellows also had opportunities to invite expert scientists to support training and research locally. For example, as part of a visiting award, the translational immunology group led by Damalie Nakanjako (Physician) invited an expert in multicolour flowcytometry (Glenda Canderan from the Department of Pathology at Case Western Reserve University, USA) to support the team through their large flowcytometry panels using the 20-color LSRII flowcytometer at UVRI campus. Subsequently the team published important results that demonstrated persistent innate immune dysfunction (NK cells, monocytes and Innate lymphoid cells) despite long-term antiretroviral therapy ^23– 25^. Similarly, maternal health group, led by Annettee Nakimuli (Obstetrician and Gynaecology), through Ashley Moffet and Corina Alberg provided several opportunities for Uganda’s junior scientists to network and collaborate with experts in the field of KIR genes and their role in pre-eclampsia which is the leading cause of poor maternal and fetal outcomes in Uganda ^26, 39^. Subsequently two more PhD students are being trained in this field on the programme. In addition, Makerere University graduate trainees (MSc and doctoral) and staff in Immunology and Bioinformatics attend regular remote seminars provided by Cambridge University using the WebEx (Cisco WebEx, Milpitas, CA).

Each of MUII’s PhD and post-doctoral Fellows had the opportunity to travel as they had to have an international collaborator as well as local collaborators. That approach multiplied the collaborations by the number of fellows. In a survey of a cancer research capacity building programme, training doctoral students and fellows, only 3/38 (7%) had adequate opportunities to network with other researchers ^37^. Our involvement of world-class experts in the fields of interest helped to mitigate the previously noted challenge of limited experts to mentor emerging scientists in Uganda ^40, 41^. Collaborators/co-supervisors and co-mentors were key elements in the programme because of the limited critical mass of mid-and-advanced career scientists in the country. In the past, many of Uganda’s experts were scattered globally due to cultural, geopolitical and socio-economic drivers of brain drain ^37, 38, 41^. Fellows highlighted that MUII provided them a lot of exposure that opened several opportunities to pursue careers in research. The multi-disciplinary nature of the collaborations established by MUII has birthed long-term bonds with the participating institutions of LSHTM, Cambridge University and the Sanger Institute, among others; which will continue to be utilised by students and faculty beyond the MUII funding. A limitation of this study is the low survey response of 40% which could bias our findings if most of the responders had been successful trainees; however, this may not affect our results because we received both positive and negative reviews in FGDs and reached saturation of ideas with this sample size. We also found that emerging themes from the trainees in FGDs were complementary to those from key informant interviews.

## Conclusion

MUII demonstrated a decade of mentored academic productivity in I&I research. Its sustainability is evidenced by the continuing mentorship, research groups, training programmes, resource mobilisation and global collaborations that have emerged to contribute to solutions to Africa’s health care problems. More skills and commitment remain necessary for trainees in developing countries to compete favourably for international research funding to build and sustain a critical mass of I&I scientists, to respond to current and emerging health problems.

## Data availability

### Underlying data

Interview transcripts contain information that could be used to identify participants. Therefore, these data are not openly available. Readers or reviewers who wish to access the data should email Damalie Nakanjako ( dnakanjako@gmail.com) or Flavia Zalwango ( Flavia.Zalwango@mrcuganda.org) to apply for access to the underlying data. Data access will be provided to researchers for the purposes of re-analysis.

### Extended data

Figshare: Interview Guide MUII_ Staff_F1000AAS.pdf. https://doi.org/10.6084/m9.figshare.12315431.v1 ^42^.

This file contains the interview guide used in the present study.

Extended data are available under the terms of the Creative Commons Attribution 4.0 International license (CC-BY 4.0).

## Author contributions

DN, FZ, PW, FL, VDB, MGM, SC, AE, contributed to the design and implementation of the MUII programme activities including the annual reports. FZ was the independent social scientist who collected data from MUII staff and trainees. DN and FZ drafted the manuscript. All authors reviewed and approved the manuscript for publication.

## References

[1] Cose S Bagaya B Nerima B: Immunology in Africa. *Trop Med Int Health.* 2015;20(12):1771–7. 10.1111/tmi.12599 26391634 PMC4737115

[2] Elliott A Nerima B Bagaya B: Capacity for science in sub-Saharan Africa. *Lancet.* 2015;385(9986):2435–7. 10.1016/S0140-6736(15)61111-4 26122054

[3] Manabe YC Nambooze H Okello ES: Group Mentorship Model to Enhance the Efficiency and Productivity of PhD Research Training in Sub-Saharan Africa. *Ann Glob Health.* 2018;84(1):170–175. 10.29024/aogh.25 30873808 PMC6748251

[4] Mullan F Frehywot S Omaswa F: The Medical Education Partnership Initiative: PEPFAR's effort to boost health worker education to strengthen health systems. *Health Aff (Millwood).* 2012;31(7):1561–72. 10.1377/hlthaff.2012.0219 22778346

[5] Sewankambo N Tumwine JK Tomson G: Enabling dynamic partnerships through joint degrees between low- and high-income countries for capacity development in global health research: experience from the Karolinska Institutet/Makerere University partnership. * PLoS Med.* 2015;12(2):e1001784. 10.1371/journal.pmed.1001784 25646629 PMC4315568

[6] Bayigga L Kateete DP Anderson DJ: Diversity of vaginal microbiota in sub-Saharan Africa and its effects on HIV transmission and prevention. *Am J Obstet Gynecol.* 2019;220(2):155–166. 10.1016/j.ajog.2018.10.014 30321529 PMC10715630

[7] Biaro IA Egesa M Kimuda S: Effect of isoniazid preventive therapy on immune responses to *mycobacterium tuberculosis*: an open label randomised, controlled, exploratory study. *BMC Infect Dis.* 2015;15:438. 10.1186/s12879-015-1201-8 26493989 PMC4619204

[8] Biraro IA Kimuda S Egesa M: The Use of Interferon Gamma Inducible Protein 10 as a Potential Biomarker in the Diagnosis of Latent Tuberculosis Infection in Uganda. *PLoS One.* 2016;11(1):e0146098. 10.1371/journal.pone.0146098 26771653 PMC4714877

[9] Egesa M Lubyayi L Jones FM: Antibody responses to *Schistosoma mansoni* schistosomula antigens. *Parasite Immunol.* 2018;40(12):e12591. 10.1111/pim.12591 30239012 PMC6492298

[10] Egesa M Lubyayi L Tukahebwa EM: *Schistosoma mansoni* schistosomula antigens induce Th1/Pro-inflammatory cytokine responses. *Parasite Immunol.* 2018;40(12):e12592. 10.1111/pim.12592 30239006 PMC6492251

[11] Kateete DP Kamulegeya R Kigozi E: Frequency and patterns of second-line resistance conferring mutations among MDR-TB isolates resistant to a second-line drug from eSwatini, Somalia and Uganda (2014-2016). *BMC Pulm Med.* 2019;19(1):124. 10.1186/s12890-019-0891-x 31291943 PMC6617586

[12] Kateete DP Nakanjako R Okee M: Genotypic diversity among multidrug resistant *Pseudomonas aeruginosa* and *Acinetobacter* species at Mulago Hospital in Kampala, Uganda. *BMC Res Notes.* 2017;10(1):284. 10.1186/s13104-017-2612-y 28705201 PMC5513047

[13] Kimuda SG Andia-Biraro I Egesa M: Use of QuantiFERON®-TB Gold in-tube culture supernatants for measurement of antibody responses. *PLoS One.* 2017;12(11):e0188396. 10.1371/journal.pone.0188396 29161328 PMC5697869

[14] Kimuda SG Biraro IA Bagaya BS: Characterising antibody avidity in individuals of varied *Mycobacterium tuberculosis* infection status using surface plasmon resonance. *PLoS One.* 2018;13(10):e0205102. 10.1371/journal.pone.0205102 30312318 PMC6185725

[15] Kimuda SG Nalwoga A Levin J: Humoral Responses to Rv1733c, Rv0081, Rv1735c, and Rv1737c DosR Regulon-Encoded Proteins of *Mycobacterium tuberculosis* in Individuals with Latent Tuberculosis Infection. *J Immunol Res.* 2017;2017:1593143. 10.1155/2017/1593143 28255560 PMC5309422

[16] Kiragga AN Castelnuovo B Musomba R: Comparison of methods for correction of mortality estimates for loss to follow-up after ART initiation: a case of the Infectious Diseases Institute, Uganda. *PLoS One.* 2013;8(12):e83524. 10.1371/journal.pone.0083524 24391780 PMC3877043

[17] Kiragga AN Lok JJ Musick BS: CD4 trajectory adjusting for dropout among HIV-positive patients receiving combination antiretroviral therapy in an East African HIV care centre. *J Int AIDS Soc.* 2014;17(1):18957. 10.7448/IAS.17.1.18957 25131801 PMC4136415

[18] Kitayimbwa JM Mugisha JY Saenz RA: The role of backward mutations on the within-host dynamics of HIV-1. *J Math Biol.* 2013;67(5):1111–39. 10.1007/s00285-012-0581-2 22955525 PMC4909148

[19] Kitayimbwa JM Mugisha JYT Saenz RA: Estimation of the HIV-1 backward mutation rate from transmitted drug-resistant strains. *Theor Popul Biol.* 2016;112:33–42. 10.1016/j.tpb.2016.08.001 27553875 PMC5126109

[20] Mpairwe H Namutebi M Nkurunungi G: Risk factors for asthma among schoolchildren who participated in a case-control study in urban Uganda. *eLife.* 2019;8:e49496. 10.7554/eLife.49496 31729315 PMC6914334

[21] Mpairwe H Ndibazza J Webb EL: Maternal hookworm modifies risk factors for childhood eczema: results from a birth cohort in Uganda. *Pediatr Allergy Immunol.* 2014;25(5):481–8. 10.1111/pai.12251 25171741 PMC4312885

[22] Mpairwe H Webb EL Muhangi L: Anthelminthic treatment during pregnancy is associated with increased risk of infantile eczema: randomised-controlled trial results. *Pediatr Allergy Immunol.* 2011;22(3):305–12. 10.1111/j.1399-3038.2010.01122.x 21255083 PMC3130136

[23] Nabatanzi R Bayigga L Cose S: Monocyte Dysfunction, Activation, and Inflammation After Long-Term Antiretroviral Therapy in an African Cohort. *J Infect Dis.* 2019;220(9):1414–1419. 10.1093/infdis/jiz320 31323092 PMC6761975

[24] Nabatanzi R Bayigga L Cose S: Aberrant natural killer (NK) cell activation and dysfunction among ART-treated HIV-infected adults in an African cohort. *Clin Immunol.* 2019;201:55–60. 10.1016/j.clim.2019.02.010 30817998 PMC6448528

[25] Nabatanzi R Cose S Joloba M: Effects of HIV infection and ART on phenotype and function of circulating monocytes, natural killer, and innate lymphoid cells. *AIDS Res Ther.* 2018;15(1):7. 10.1186/s12981-018-0194-y 29544508 PMC5853105

[26] Nakimuli A Chazara O Hiby SE: A *KIR B* centromeric region present in Africans but not Europeans protects pregnant women from pre-eclampsia. *Proc Natl Acad Sci U S A.* 2015;112(3):845–50. 10.1073/pnas.1413453112 25561558 PMC4311823

[27] Nampijja M Apule B Lule S: Effects of maternal worm infections and anthelminthic treatment during pregnancy on infant motor and neurocognitive functioning. *J Int Neuropsychol Soc.* 2012;18(6):1019–30. 10.1017/S1355617712000768 23158229 PMC3948080

[28] Nampijja M Webb E Nanyunja C: Randomised controlled pilot feasibility trial of an early intervention programme for young infants with neurodevelopmental impairment in Uganda: a study protocol. *BMJ Open.* 2019;9(10):e032705. 31601606 10.1136/bmjopen-2019-032705 PMC6797334

[29] Sanya RE Webb EL Zziwa C: The effect of helminth infections and their treatment on metabolic outcomes: results of a cluster-randomised trial. *Clin Infect Dis.* 2020;71(3):601–613. 10.1093/cid/ciz859 31504336 PMC7384320

[30] Tweyongyere R Mawa PA Emojong NO: Effect of praziquantel treatment of *Schistosoma mansoni* during pregnancy on intensity of infection and antibody responses to schistosome antigens: results of a randomised, placebo-controlled trial. *BMC Infect Dis.* 2009;9:32. 10.1186/1471-2334-9-32 19296834 PMC2666740

[31] Tweyongyere R Namanya H Naniima P: Human eosinophils modulate peripheral blood mononuclear cell response to Schistosoma mansoni adult worm antigen *in vitro*. *Parasite Immunol.* 2016;38(8):516–22. 10.1111/pim.12336 27169695 PMC4973678

[32] Tweyongyere R Naniima P Mawa PA: Effect of maternal *Schistosoma mansoni* infection and praziquantel treatment during pregnancy on *Schistosoma mansoni* infection and immune responsiveness among offspring at age five years. *PLoS Negl Trop Dis.* 2013;7(10):e2501. 10.1371/journal.pntd.0002501 24147175 PMC3798616

[33] Manabe YC Katabira E Brough RL: Developing independent investigators for clinical research relevant for Africa. *Health Res Policy Syst.* 2011;9:44. 10.1186/1478-4505-9-44 22206247 PMC3283488

[34] Shen C Wang Z Zhao F: Treatment of 5 Critically Ill Patients With COVID-19 With Convalescent Plasma. *JAMA.* 2020;323(16):1582–1589. 10.1001/jama.2020.4783 32219428 PMC7101507

[35] Spellberg B Haddix M Lee R: Community Prevalence of SARS-CoV-2 Among Patients With Influenzalike Illnesses Presenting to a Los Angeles Medical Center in March 2020. *JAMA.* 2020;323(19):1966–1967. 10.1001/jama.2020.4958 32232421 PMC7110920

[36] Anthony F: Clinical Conversations: Anthony Fauci on Talking with Patients About COVID-19. *NEJM Journal Watch.* 2020 Reference Source

[37] Phipps W Kansiime R Stevenson P: Peer Mentoring at the Uganda Cancer Institute: A Novel Model for Career Development of Clinician-Scientists in Resource-Limited Settings. *J Glob Oncol.* 2018;4:1–11. 10.1200/JGO.17.00134 30241258 PMC6223430

[38] Nakanjako D Katamba A Kaye DK: Doctoral training in Uganda: evaluation of mentoring best practices at Makerere university college of health sciences. *BMC Med Educ.* 2014;14:9. 10.1186/1472-6920-14-9 24410984 PMC3897930

[39] Blokhuis JH Hilton HG Guethlein LA: KIR2DS5 allotypes that recognize the C2 epitope of HLA-C are common among Africans and absent from Europeans. *Immun Inflamm Dis.* 2017;5(4):461–468. 10.1002/iid3.178 28685972 PMC5691316

[40] Nakanjako D Akena D Kaye DK: A need to accelerate health research productivity in an African University: the case of Makerere University College of Health Sciences. *Health Res Policy Syst.* 2017;15(1):33. 10.1186/s12961-017-0196-6 28431554 PMC5399829

[41] Nakanjako D Byakika-Kibwika P Kintu K: Mentorship needs at academic institutions in resource-limited settings: a survey at Makerere University College of Health Sciences. *BMC Med Educ.* 2011;11:53. 10.1186/1472-6920-11-53 21801406 PMC3170866

[42] Nakanjako D: Interview Guide MUII_ Staff_F1000AAS.pdf. *figshare.*Journal contribution.2020 10.6084/m9.figshare.12315431.v1

