# Supplementary material for: Career development for infection and immunity research in Uganda: a decade of experience from the Makerere University – Uganda Virus Research Institute research and training programme
Source: AAS Open Res. 2020 Aug 17;3:26. Originally published 2020 Jun 24. [Version 2] doi: 10.12688/aasopenres.13066.2 (PMC7372530; doi:10.12688/aasopenres.13066.2)
Supplement: Peer review history

# Peer review: Reviewer response for version 1

**DOI:** 10.21956/aasopenres.14161.r27508

**Published:** 2020-07-20

**Authors:** Allen Muyaama Mukhwana

**Affiliations:** 1 African Academy of Sciences (AAS), Nairobi, Kenya

I have read the manuscript, it is well written. Having said that, below are some suggestions to improve the article;  

1. In the introduction the authors mention post-doctoral and group leaders as early-career scientists, it would be of benefit to explain the difference between these two categories.
2. Methods: The authors mention a secretariat and an executive committee (both headed by the director) managing the implementation of the programme. Also mentioned is an efficient administration that provides an enabling environment.
   
  1. What is the enabling environment?
  2. What is the role of secretariat if the management of the programme is done by the executive committee?
  3. It would be helpful to understand the difference in the roles above and why? What are the successes, changes and key lessons learned from this arrangement?
  4. How the two (in b above) that separated from the UVRI administrative structure mentioned in table 2?
  5. The authors can explain the limitations of purposefully selecting the 27 trainees from the small pool of 68 trainees.
3.  Both EMEL029, F, IDI, KII, Centre Staff & EMEL018, F, IDI, Group Leader mention Obs and Gyn Department and mentoring of gynaecologists, the authors have not mentioned this group anywhere else.   
4. Table 2:
   
  1. As MUII’s contribution to laboratory capacity is ‘technology transfer by fellows training abroad’ – what is the authors’ definition of ‘technology transfer’?
  2. Please expound on the “…human, technical and physical infrastructural development in I&I capacity” that MUII has contributed to at partner institutions.
  3. Research leadership and developing collaborations & networks are not categorized as scientific citizenship under DELTAS Africa.
5. Results: the authors have described success and career progression of trainees.  
   
  1. It would be informative to show the number of fellows that grew through the ranks (from masters through to early career).
  2. How long does it take to grow I&I scientists for public utility? Describe the support referred to under mentorship.
  3. Is the growth and success of the fellows in leadership roles totally attributed to MUII?  
6. Under sustainable training and research collaboration the authors mention fields in which world-class expertise was not available in Uganda – is there evidence for this? Was the expertise limited or totally not available?

 

Is the work clearly and accurately presented and does it cite the current literature?

Yes

If applicable, is the statistical analysis and its interpretation appropriate?

Yes

Are all the source data underlying the results available to ensure full reproducibility?

Yes

Is the study design appropriate and is the work technically sound?

Yes

Are the conclusions drawn adequately supported by the results?

Yes

Are sufficient details of methods and analysis provided to allow replication by others?

Partly

Reviewer Expertise:

Research management and governance with over ten tens years managing capacity-building programmes at research institutions.

I confirm that I have read this submission and believe that I have an appropriate level of expertise to confirm that it is of an acceptable scientific standard.

# Peer review: Reviewer response for version 1

**DOI:** 10.21956/aasopenres.14161.r27510

**Published:** 2020-07-02

**Authors:** Yohana Mashalla

**Affiliations:** 1 Department of Biomedical Sciences, Faculty of Medicine, University of Botswana, Gaborone, Botswana

I have read the manuscript thoroughly and the study has scientific basis and the document is well written. However, below are some of the improvements that can be made:

1. The introduction is rather brief and some of the information presented under the discussion can be useful to inform the introduction.
2. The methods:
  - The methods are well described. The authors should consider describing how consistency and trustworthiness of the data was ensured in this study. Such information is important in qualitative studies.
  - The authors have described (under qualitative data management) that data was analysed thematically. A table indicating the major themes and sub-themes generated and also the meaning of each would add value to the manuscript. Clear definitions of themes and subthemes would enhance understanding the description under results, the quotations as evidence on the themes.
3. Results:
  - The characteristics of respondents will add value to the manuscript of a table on demographic characteristics of the respondents could be provided. Table 1 is all about the achievements rather than who provided the information.
  - It appears from the results that only two major themes have been discussed i.e. achievements and challenges. This section should be structured to reflect the major themes cited under methods. Introduce sub-heading e.g. Achievements, Good practices, lesson learnt, recommendations for improvements etc.
4. Discussion:
  - The discussion is well written but paragraph 5 is probably best placed under methods. This paragraph discusses establishment of a secretariat, an operational overseeing structure, therefore not part of the results.
  - The discussion should provide some of the recommendations made by respondents.
5. Graphs and tables:
  The tables available are well presented. Check figure 2(B) on the y-axis, the UK Sterling Pound is written in plural (Pounds).
6. Typo/grammatical errors:
  Check under "Data availability", line 4, after the name "Nakanjako" there is Flavia Zalwango. Delete because it is repeated on the next line 5.

Is the work clearly and accurately presented and does it cite the current literature?

Yes

If applicable, is the statistical analysis and its interpretation appropriate?

Yes

Are all the source data underlying the results available to ensure full reproducibility?

Yes

Is the study design appropriate and is the work technically sound?

Yes

Are the conclusions drawn adequately supported by the results?

Yes

Are sufficient details of methods and analysis provided to allow replication by others?

Partly

Reviewer Expertise:

A medical doctor, clinical respiratory physiologist who in the past ten years has engaged in global health initiatives specifically training health professionals (doctors, nurses, public health specialists) to become effective global health leaders.

I confirm that I have read this submission and believe that I have an appropriate level of expertise to confirm that it is of an acceptable scientific standard.
